# Supplementary material for: Validation of Food Compass with a healthy diet, cardiometabolic health, and mortality among U.S. adults, 1999–2018
Source: Nat Commun. 2022 Nov 22;13:7066. doi: 10.1038/s41467-022-34195-8 (PMC9681774; doi:10.1038/s41467-022-34195-8)
Supplement: Supplementary file 1 — Supplementary Information [file 41467_2022_34195_MOESM1_ESM.pdf]

Supplementary Materials

Supplementary Tables

Table S1. Domains and attributes of the Food Compass Score.
Table S2. Scoring principles for the 56 attributes within 9 domains of the Food Compass
Table S3. Defining prevalent health conditions
Table S4. Population characteristics of U.S. adults by i.FCS quintile, 1999-2018
Table S5. Count and percentage of energy intake (median [IQR]) of reported foods and beverage products corresponding to FCS ≤30, 31-69, and ≥70 thresholds by i.FCS
Table S6. Relationship between Healthy Eating Index (HEI) 2015 and the individual Food Compass Score (i.FCS) among U.S. adults, 1999-2018, by population subgroup.
Table S7. Sensitivity analysis of the multivariable adjusted, survey-weighted cross-sectional analysis of the association between individual Food Compass Score (i.FCS) and cardiometabolic health among U.S. adults, 1999-2018
Table S8. Multivariable adjusted, survey-weighted prospective analysis of individual Food Compass Score and the 9 components of the individual Food Compass Score with all-cause and cause-specific mortality among U.S. adults, 1999-2014
Table S9. Sensitivity analysis of the multi-variable adjusted, survey-weighted prospective association between individual Food Compass Score and all-cause and cause-specific mortality among U.S. adults, 1999-2018, censoring at 85+ years and incorporating additional covariates
Table S10. Multivariable adjusted, survey-weighted prospective association between individual Food Compass Score (i.FCS) and all-cause mortality, stratified by key sociodemographic factors, among U.S. adults, 1990-2018
Table S11. Missing values for total flavonoids, vitamin D, and choline in FNDDS 2001-2018
Table S12. Comparison of modified vs. original Food Compass algorithm target low and high scores for food ingredients domain attributes
Table S13. Survey-weighted mean total physical activity (MET-hours/week) calculated based on imputation among U.S. adults, 1999 -2018
Table S14. Survey-weighted mean total physical activity (MET-hours/week) without imputation among U.S. adults, 1999 -2018
Table S15. Survey-weighted mean leisure-time physical activity (MET-hours/week) among U.S. adults, 1999 -2018
Table S16. Missing values for clinical risk factors and health conditions in NHANES, 1999-2018

Supplementary Figures

Figure S1. Flow diagram for inclusion of NHANES participants in cross-sectional and mortality analysis.
Figure S2. Relationship between Healthy Eating Index (HEI) 2015 and the 9 components of the individual Food Compass Score (i.FCS) among U.S. adults, 1999-2018.
Figure S3. Semi-parametric, restricted cubic spline analysis testing non-linearity in the association between individual Food Compass Score and all-cause mortality among U.S. adults, 1999-2014.

Supplementary Notes

Note 1. Identification of complete and reliable dietary recall observations in NHANES, 1999-2018
Note 2. Missing attribute values in FNDDS 2001-2018 for nutrient profiling
Note 3. Modifications to the Food Compass algorithm
Note 4. Development of total physical activity (MET-hours/week) variable in NHANES, 1999-2018
Note 5. Missing values for clinical risk factors and health conditions in NHANES, 1999-2018.

Table S1. Domains and attributes of the Food Compass Score.*

Nutrient Ratios	Vitamins (top 5)	Minerals (top 5)	Food-based Ingredients	Additives	Processing	Specific Lipids (0.5 weight) (top 3)	Fiber & Protein (0.5 weight)	Phytochemicals (0.5 weight)
Unsaturated: Saturated fat ratio	Vitamin A	Calcium	Fruits (non-dried & dried)	Added sugar	NOVA classification	ALA	Total fiber	Total flavonoids
Fiber: Carbohydrate ratio†	Thiamin (B1)	Phosphorus	Vegetables, non-starchy (non-dried, and dried)	Nitrites	Fermentation	EPA + DHA	Total protein	Total carotenoids
Potassium: Sodium ratio†	Riboflavin (B2)	Magnesium	Fruit juice	Artificial sweeteners, flavors, or colors§	Frying	Medium-chain fatty acids (MCFA)		
	Niacin (B3)	Iron	Vegetable juice	Partially hydrogenated oils‡§		Dietary cholesterol		
	Vitamin B6	Zinc	Beans & legumes	Interesterified or hydrogenated oils§		Trans fats§		
	Folate (B9)	Copper	Whole grains	High fructose corn syrup§				
	Cobalamin (B12)	Selenium	Nuts and seeds	Monosodium glutamate (MSG) §				
	Vitamin C	Sodium	Seafood					
	Vitamin D	Potassium	Yogurt					
	Vitamin E	Iodine§	Plant oils					
	Vitamin K		Refined grains					
	Choline		Red or processed Meat					

*The Food Compass incorporates 56 individual attributes across 9 domains, each assessed per 100 kcal (418.4 kj) of food product. Attributes are scored across a 10-point range (0 to 10 for beneficial factors; -10 to 0 for harmful factors; and -10 to 10 for nutrient ratios which could range from harmful to beneficial). Attributes with emerging evidence for health impacts, including 5 additives and fermentation, were scored using half weights. Each domain then received a score, calculated as the average of all attributes in that domain (or for food ingredients, as the sum, given contents of ingredients are interdependent). For 3 domains (vitamins, minerals, specific lipids), the highest (absolute value, i.e. negative or positive) 5, 5, and 3 scores, respectively, were calculated. First, many foods with health impacts are rich in one or just a few, but not numerous, vitamins, minerals, or specific lipids. Thus, averaging attribute scores across all 12 vitamins, for example, would incorporate many very low or zero values, causing this domain to be scored more similarly for most foods and weakening its discrimination of healthful foods with naturally higher levels of a few vitamins. Second, at the other extreme, the scoring approach for these domains constrained the potential impact of excessive fortification of multiple different vitamins, minerals, or specific lipids during processing. All domains

scores were then summed, using equal weights for the first 6 domains and half weights for the latter 3 domains. The final Food Compass score (FCS) was scaled across all food and beverage items to range from 1 (least healthful) to 100 (most healthful). For full scoring details of each attribute and the scaling of the final sum, see Table S2.

† Potassium and sodium were included in the Food Compass both as Nutrient Ratios given evidence for their biologic interaction, and separately as Minerals given evidence that their absolute intakes also influence health. Carbohydrate and fiber were included as Nutrient Ratios given evidence that their ratio predicts healthfulness of carbohydrate-rich foods; and separately as total fiber (in Fiber & Protein) given evidence that the absolute intake of dietary fiber, but not total carbohydrate, influences health.

‡ Partially hydrogenated oils (emerging, scored with a half-weight) were included in addition to trans fats content (scored -10 to 0 in Specific Lipids) based on emerging evidence that industrial trans fats may have greater adverse health effects than naturally occurring trans fats; and that presence of such partially hydrogenated oils may serve as a marker of more intensive and potentially adverse industrial processing.

§ Iodine, artificial sweeteners, flavors, or colors, partially hydrogenated oils, interesterified or hydrogenated oils, high fructose corn syrup, monosodium glutamate, and trans fats were excluded from scoring in this analysis, as these attributes are not available within in the available USDA databases used (FNDDS, FPED, flavonoid database).

AI, adequate intake. ALA, alpha-linolenic acid. EPA + DHA, eicosapentaenoic acid + docosahexaenoic acid. UL, upper limit intake.

Table S2. Scoring principles for the 56 attributes within 9 domains of the Food Compass.*

Attributes (per 100 kcal [418.4 kj])	Attribute Points	Target for low score	Target for high score	Target Reference†
**Nutrient Ratios ‡**				
Unsaturated:Saturated fat ratio (log)	-10 to 10	-0.66	1.77	5th, 95th pctl
Fiber:Carbohydrate ratio (log)	-10 to 10	-7.02	-0.78	5th, 95th pctl
Potassium:Sodium ratio (log)	-10 to 10	-2.02	3.30	5th, 95th pctl
**Vitamins (top 5) §**				
Vitamin A, RAE	0 to 10	0	225 ug	25% RDA
Thiamin (B1)	0 to 10	0	0.3 mg	25% RDA
Riboflavin (B2)	0 to 10	0	0.325 mg	25% RDA
Niacin (B3)	0 to 10	0	4 mg	25% RDA
Vitamin B-6	0 to 10	0	0.325 mg	25% RDA
Folate, DFE (B9)	0 to 10	0	100 ug	25% RDA
Cobalamin (B12)	0 to 10	0	0.6 ug	25% RDA
Vitamin C	0 to 10	0	22.5 mg	25% RDA
Vitamin D (D2 + D3)	0 to 10	0	3.75 ug	25% RDA
Vitamin E (alpha-tocopherol)	0 to 10	0	3.75 mg	25% RDA
Vitamin K (phylloquinone)	0 to 10	0	30 ug	25% AI
Choline, total	0 to 10	0	137.5 mg	25% AI
**Minerals (top 5) §**				
Calcium	0 to 10	0	250 mg	25% RDA
Phosphorus	0 to 10	0	175 mg	25% RDA
Magnesium	0 to 10	0	105 mg	25% RDA
Iron	0 to 10	0	4.5 mg	25% RDA
Zinc	0 to 10	0	2.75 mg	25% RDA
Copper	0 to 10	0	0.225 mg	25% RDA
Selenium	0 to 10	0	13.75 ug	25% RDA
Sodium	-10 to 0	575 mg	0	25% RDA
Potassium	0 to 10	0	1175 mg	25% RDA
Iodine	0 to 10	0	37.5 ug	25% RDA
**Food Ingredients (sum) ‖**				
Fruits	0 to 10	0	1.75 cups	95th pctl
Fruits, dried	0 to 10	0	0.75 cups	95th pctl
Fruit juice	0 to 5	0	1.08 cups	95th pctl
Vegetables, non-starchy	0 to 10	0	4.77 cups	95th pctl
Vegetables, non-starchy & dried	0 to 10	0	2.41 cups	95th pctl
Vegetable juice	0 to 5	0	2.41 cups	95th pctl
Beans and legumes	0 to 10	0	0.50 cups	95th pctl
Whole grains	0 to 10	0	1.12 oz	95th pctl
Nuts and seeds	0 to 10	0	1.35 oz	95th pctl
Seafood	0 to 10	0	3.86 oz	95th pctl
Yogurt	0 to 10	0	0.81 cups	95th pctl
Plant Oils	0 to 10	0	11.31 g	95th pctl
Refined grains	-10 to 0	1.45 oz	0	95th pctl
Red or processed meat	-10 to 0	2.69 oz	0	95th pctl
**Additives ¶**				

Attributes (per 100 kcal [418.4 kj])	Attribute Points	Target for low score	Target for high score	Target Reference†
Added sugar, % calories	-10 to 0	100%	0	
Nitrites, % calories from processed meats	-10, 0	25%	0	
Artificial sweeteners, flavors, or colors	-10, 0	yes	no	
Partially hydrogenated oils	-10, 0	yes	no	
Interesterified or hydrogenated oils	-10, 0	yes	no	
High fructose corn syrup	-10, 0	yes	no	
Monosodium glutamate (MSG)	-10, 0	yes	no	
**Processing €**				
NOVA processing level	-10, -2, -1, 0	NOVA=4	NOVA=1	
Fermentation, % calories from fermented foods	0, 10	0	50%	
Frying	-10, 0	yes	no	
**Specific Lipids (top 3) ¥**				
Cholesterol	-10 to 0	75 mg	0	25% DGA
MCFAs (8:0 + 10:0 + 12:0, g)	0 to 10	0	0.32 g	95th pctl
ALA (18:3n-3)	0 to 10	0	0.4 g	25% AI
EPA + DHA (20:5n-3 + 22:6n-3)	0 to 10	0	62.5 mg	25% REC
Trans fats, % calories	-10 to 0	30%	0	expert consensus
**Fiber & Protein §**				
Total fiber	0 to 10	0	9.5 g	25% AI
Total protein	0 to 10	0	14 g	25% RDA
**Phytochemicals §**				
Total flavonoids	0 to 10	0	23.53 mg	95th pctl
Total carotenoids	0 to 10	0	8746.81 mcg	95th pctl

* Each domain score was calculated as the average of the attribute scores in that domain (or the sum for the food ingredient domain), and the 9 domain scores were summed (with half-weights for specific lipids, fiber & protein, and phytochemicals). To minimize the influence outliers, the range of summed scores across all 58622 scored food and beverage items (FNDDS 2001-2018) was first truncated at the 5th and 95th percentiles (-10.4, 27.2). The final Food Compass Score (FCS) was then scaled across all food and beverage items to range from 1 (least healthful) to 100 (most healthful) using the equation: FCS = [100 – (((max score – unscaled score)/score range))*99)], or [100 – (((27.2– unscaled score)/37.7))*99)].

† When Dietary Reference Intakes (DRIs) varied by population subgroup, the DRI was selected for adults age 19-50 years (and for men when varying by sex). Across attributes, the 25% threshold for DRIs was identified as most consistently distinguishing foods with higher vs. lower levels of these nutrients, and was generally similar to the 95% percentile value of content across all foods and beverages reported in NHANES 2015-16. For attributes without DRIs, scoring was based on the distributions of relevant, unique food and beverage items reported in FNDDS 2001-2018. Whether scoring should remain constant for other datasets and nations, or vary according to the local food items, is a topic for further investigation.

‡ Nutrient ratios were each scored on a log-linear scale from -10 to 10. To prevent excessive scoring influence of small amounts of these nutrients in any food item, the unsaturated: saturated fat ratio was not calculated for items with <10% energy from fat; the fiber: carbohydrate ratio, for items with <10% energy from carbohydrate; and potassium: sodium ratio, for items with <0.01 mg of either potassium or sodium per 100 kcal. Absolute levels of fiber were also separately included under Fiber & Protein; and of potassium and sodium, under Minerals; in order to capture their separate health effects independent of these interactions represented by the ratios.

§ Vitamins, minerals, fiber, protein, flavonoids, and carotenoids were each scored on a linear scale from 0 to 10.

‖ Food-based ingredients were each scored on a linear scale from 0 to 10 for healthful ingredients; 0 to 5 for fruit and vegetable juices; and from -10 to 0 for harmful ingredients (refined grains, red or processed meat). The 95th percentile for each food-based ingredient was calculated based on a subset of relevant foods assessed (i.e. yogurt for the food-based yogurt attribute). Separate 95th percentile values were calculated and used for dried vs. non-dried fruits and dried vs. non-dried, non-starchy vegetables given

their different water weights. The attribute scores in this domain were summed (not averaged), because contents of each ingredient are mutually interdependent.

¶ Because added sugar content is nonlinear (skewed) across the food supply, it was scored as follows: 0 (<2% calories), -1 (2-3% calories), -2 (4-5% calories), -3 (6-7% calories), -4 (8-9% calories), -5 (10-19% calories), -6 (20-39% calories), -7 (40-59% calories), -8 (60-79% calories), -9 (80-99%), and -10 (100%). As no threshold has been set for nitrate content, nitrites were scored using percentage energy from processed/cured meats as a proxy. 25% energy from processed/cured meat was assigned the target low score (-10), with linear scaling down to 0% energy from processed/cured meats as the target high score (0). For calculating the domain score, added sugar and nitrites, considered to have stronger evidence for health harms, were each provided a full weight, while the remaining additives, considered to have emerging and less robust evidence for independent health harms based on more limited studies, were each provided a half weight.

€ The NOVA processing classification was scored as -10, -5, -2.5, and 0 for the 4 categories of ultra-processed, processed, culinary ingredients, and un-processed/minimally foods, respectively. For mixed dishes (about 2/3 of all products), NOVA classification was calculated based on an energy-weighted score of the constituent ingredients' NOVA classification, leading to non-integer NOVA classification values. As such, attribute scores for NOVA classification were linearly scaled between the integer NOVA classes 1-4. Fermentation was defined based on energy content from yogurt or cheese. 50% energy from yogurt + cheese was assigned the target high score (10), with linear scaling down to 0% energy from yogurt or cheese as the target low score (0). Additionally, other fermented products were identified by keyword search (i.e., kefir, kombucha, injera, dosa, natto, miso, kimchi, etc.) and assigned the target high score. Frying was defined based on the name and/or cooking description of each food item (e.g., "fried catfish"). For calculating the domain score, the NOVA classification system, considered to have stronger evidence for health harms, was provided a full weight, while fermentation and frying, considered to have emerging and less robust evidence for independent health harms based on more limited studies, were each provided a half weight.

¥ Specific lipids were each scored on a linear scale from 0 to 10 (ALA, EPA+DHA, MCFA) or -10 to 0 (cholesterol, trans fats). For calculating the domain score, MCFA content was considered to have emerging and less robust evidence for independent health harms based on more limited studies, and was provided a half weight.

AI, adequate intake. ALA, alpha-linolenic acid. EPA + DHA, eicosapentaenoic acid + docosahexaenoic acid. MCFAs, medium-chain fatty acids. RDA, recommended daily allowance. REC, dietary recommendation. UL, upper limit intake

Table S3. Defining prevalent health conditions

Prevalent health condition	Definition
Metabolic syndrome*	Any three of the following criteria:<br>1) Waist circumference: ≥88 cm (women) | ≥102 cm (men)<br>2) Triglycerides: ≥150 mg/dL or on lipid-lowering medication<br>3) HDL-C: <50 mg/dL (women) | <40 mg/dL (men) or on lipid-lowering medication<br>4) Blood pressure: SBP ≥130 mm Hg or ≥ 85 mmHg diastolic or on blood pressure lowering medication<br>5) Fasting plasma glucose: ≥ 100 mg/dL or on diabetes medications
Diabetes mellitus†	At least one of the following:<br>1) HbA1c: > 6.5%<br>2) Fasting plasma glucose: > 126 mg/dL<br>3) Self-reported use of diabetes medication
Cardiovascular disease	Presence of both of the following:<br>1) Self-reported history of coronary heart disease, heart failure, stroke, myocardial infarction, or angina ‡<br>2) Self-reported use of angina, hypertension, or lipid-lowering medication
Cancer	Self-reported history of cancer of any form
Lung disease	Self-reported history of at least one of the following:<br>1) Emphysema<br>2) Bronchitis<br>3) Asthma<br>4) Chronic obstructive pulmonary disease (COPD)
Optimal cardiometabolic health	All of the following criteria:<br>1) Adiposity: BMI <25 kg/m² AND WC ≤88 cm [women] | ≤102 cm [men]<br>2) Blood glucose: FPG <100 mg/dL and HbA1c <5.7% and not taking diabetes medication<br>3) Blood lipids: total cholesterol: HDL ratio <3.5:1 and not taking lipid lowering medication §<br>4) Blood pressure: SBP < 120 mmHg, DBP < 80 mmHg and not taking blood pressure medication ‖<br>5) History of CVD: no self-reported CHD, heart failure, myocardial infarction, stroke or angina ‡

* Standard AHA/NHLBI definition of metabolic syndrome[1]

† FPG and HbA1c levels from the American Diabetes Association.[2] FPG values prior to 2015-16 NHANES survey cycles were corrected to account for differences in measurement instruments used.

‡ Presence of angina based on having both a positive Rose Questionnaire or use of anti-anginal medication.

§ TC:HDL levels from Calling *et al*.[3]

‖ Blood pressure levels from Unger *et al*.[4]

BMI, body mass index; DBP, diastolic blood pressure; FPG, fasting plasma glucose; HbA1c, hemoglobin A1c; SBP, systolic blood pressure; TC:HDL, total cholesterol to high-density lipoprotein ratio; WC, waist circumference

Table S4. Population characteristics of U.S. adults by i.FCS quintile, 1999-2018

	No. of participants (weighted %)*				
	i.FCS quintile				
Characteristics	Q1 n=9600	Q2 n=9600	Q3 n=9599	Q4 n=9600	Q5 n=9600
**Age group, y**					
20-34	3445 (24.4)	2887 (18.8)	2464 (15.5)	2013 (12.1)	1670 (10.4)
35-49	2675 (30.0)	2552 (27.9)	2427 (25.0)	2287 (23.6)	2016 (20.0)
50-64	2008 (27.6)	2174 (28.3)	2315 (31.1)	2484 (31.8)	2727 (32.8)
65+	1472 (18.1)	1987 (25.0)	2393 (28.3)	2816 (32.5)	3187 (36.8)
**Sex**					
Male	5169 (54.0)	4905 (51.1)	4595 (47.4)	4349 (43.9)	4011 (40.7)
Female	4431 (46.0)	4695 (48.9)	5004 (52.6)	5251 (56.1)	5589 (59.3)
**Race/Ethnicity**					
Mexican American	1392 (6.2)	1769 (7.6)	1892 (7.8)	1775 (6.8)	1591 (6.3)
Other Hispanic	587 (4.2)	712 (4.9)	745 (4.6)	884 (5.0)	980 (5.5)
Non-Hispanic White	4574 (70.4)	4228 (69.7)	4286 (71.6)	4424 (73.9)	4078 (70.9)
Non-Hispanic Black	2571 (14.6)	2289 (12.9)	1938 (10.0)	1616 (8.2)	1548 (7.7)
Asian/Other†	476 (4.5)	602 (5.0)	738 (6.0)	901 (6.1)	1403 (9.6)
**Education level**					
< HS graduate	2897 (23.5)	2687 (19.7)	2559 (17.7)	2436 (16.0)	2190 (13.6)
HS graduate	2794 (31.5)	2454 (28.0)	2241 (25.2)	2011 (21.8)	1638 (17.1)
Some college or AA school	2828 (30.3)	2831 (31.2)	2840 (31.0)	2628 (29.5)	2530 (27.6)
≥College graduate	1081 (14.8)	1628 (21.1)	1959 (26.1)	2525 (32.6)	3242 (41.6)
**Ratio of Family Income to Poverty †**					
<1.30	3619 (27.7)	3049 (22.2)	2892 (19.9)	2677 (18.1)	2497 (16.3)
1.30-2.49	3228 (32.7)	3240 (31.1)	3153 (30.6)	3012 (28.0)	2751 (25.0)
3.50+	2753 (39.6)	3311 (46.6)	3554 (49.5)	3911 (53.9)	4352 (58.7)
**Smoking status**					
non-smoker	4219 (42.0)	4892 (49.3)	5298 (51.1)	5619 (56.1)	6039 (59.2)
former smoker	1970 (23.5)	2277 (26.5)	2495 (30.2)	2619 (30.8)	2678 (32.1)
current smoker	3411 (34.5)	2431 (24.1)	1806 (18.7)	1362 (13.1)	883 (8.6)
**Lifestyle and metabolic health characteristics (mean [SD])**					
Average total energy intake, kcal ‡	2170 (849)	2150 (80)	2076 (78)	1967 (720)	1828 (699)
Alcohol use, % energy‡§	2.5 (6.3)	2.4 (5.7)	2.6 (5.8)	2.8 (6.0)	2.8 (6.0)
Total PA, MET-hours/week§	65.6 (109.0)	61 (107.6)	54.6 (95.9)	48.8 (89.2)	49.4 (80.8)
Leisure time PA, MET-hours/week	12.4 (34.5)	12.8 (28.8)	14.1 (28.8)	15.6 (33.3)	18 (26.7)

* Reported means and proportions reflect all sampled individuals stratified by i.FCS quintile, pooled across relevant survey cycles. NHANES dietary sample weights are incorporated to account for the complex survey design and response rates, providing nationally representative summary statistics for the non-institutionalized U.S. adult population.

† Represents the ratio of family income to the federal poverty threshold, adjusting for household size. A higher ratio indicates a higher level of income

‡ For average total energy intake (in kcal) and alcohol use (in percentage total energy), the value was calculated based on the total number of recall days available for each respondent. For example, when two days of data were available, an arithmetic average of that factor was calculated.

§ After adjusting for age (continuous) and sex, the associations for i.FCS quintile with both average total energy intake (kcal) and total physical activity (MET-hours/week) were present but attenuated as compared to the crude associations.

AA, Associates Degree; HS, high school; i.FCS, individual, dietary Food Compass Score; kcal, kilocalorie; MET, metabolic equivalent of task; PA, physical activity; SD, standard deviation

Table S5. Number and contribution to energy of specific foods and beverage products (FCS ≥70; FCS 31-69, and FCS≤30) among individual people with i.FCS ≤30, 31-69, and ≥70.*

	**i.FCS for individual persons**		
	**≤30 (n = 15,714 individuals, 32.7%)**	**31-69 (n=32,069 individuals, 66.8%)**	**≥70 (n = 216 individuals, 0.5%)**
**Contribution of food and beverage products in each FCS category †**			
Products with FCS ≤30			
Count (median, IQQ)	11 (7, 5)	8 (5, 12)	2 (1, 4)
% energy contribution (median, IQR)	65.0 (56.1, 74.4)	39.1 (28.3, 48.8)	5.9 (2.1, 10.4)
Products with FCS 31-69			
Count (median, IQQ)	5 (3, 8)	8 (5, 11)	5 (3, 8)
% energy contribution (median, IQR)	30.6 (20.8, 40.4)	44.7 (33.5, 56.0)	27.8 (18.7, 37.2)
Products with FCS ≥70			
Count (median, IQQ)	2 (1, 4)	6 (3, 9)	13 (8, 20)
% energy contribution (median, IQR)	2.3 (0.2, 6.1)	14.2 (7.3, 23.2)	65.5 (57.9, 71.4)
Total number of products reported	20	24	24

* The individual Food Compass Score (i.FCS) was calculated as the energy-weighted mean FCS of all foods and beverages consumed, as reported in 24 hr recall. The overall score could range from 1 to 100.

†The FCS for each food and beverage was calculated based on available 24 hr recall data, and categorized into 3 major categories: ≤30 for foods to limit; 31-69 for foods to consume in moderation; and ≥70 for foods to encourage.

i.FCS, individual Food Compass score; IQR, interquartile range; FCS, Food Compass Score

Table S6. Relationship between Healthy Eating Index (HEI) 2015 and the individual Food Compass Score (i.FCS) among U.S. adults, 1999-2018, by population subgroup

Sociodemographic characteristics	Spearman correlation of HEI 2015 and I.FCS
Age category, years	
20-34	0.81
35-49	0.81
50-64	0.83
65+	0.82
Sex	
Male	0.80
Female	0.82
Race/ethnicity	
Mexican-American	0.76
Other Hispanic	0.82
Non-Hispanic White	0.83
Non-Hispanic Black	0.79
Asian/Other	0.83
Education Level	
<HS graduate	0.78
HS graduate	0.81
Some college or AA school	0.81
≥College graduate	0.83
Ratio of Family Income to Poverty*	
<1.3	0.79
1.3-2.9	0.80
3+	0.83

* Represents the ratio of family income to the federal poverty threshold, adjusting for household size. A higher ratio indicates a higher level of income

AA, Associates Degree; HEI, Healthy Eating Index; HS, high school; i.FCS, individual, dietary Food Compass Score

Table S7. Sensitivity analysis of the multivariable adjusted, survey-weighted cross-sectional analysis of the association between individual Food Compass Score (i.FCS)* and cardiometabolic health among U.S. adults, 1999-2018

Continuous biomarkers†	Primary analysis	Additional covariates (total energy + survey cycle) ‡
BMI, kg/m^2	-0.60 (-0.70, -0.51)	-0.68 (-0.77, -0.59)
Systolic blood pressure, mmHg	-0.69 (-0.91, -0.48)	-0.62 (-0.83, -0.41)
Diastolic blood pressure, mmHg	-0.49 (-0.66, -0.32)	-0.46 (-0.63, -0.29)
LDL-C, mg/dL	-2.01 (-2.63, -1.40)	-1.48 (-2.11, -0.85)
HDL-C (mg/dL)	1.65 (1.44, 1.85)	1.61 (1.40, 1.81)
Triglycerides, mg/dL	-1.55 (-3.13, 0.03)	-1.09 (-2.67, 0.50)
TC: HDL ratio, 1 unit	-0.13 (-0.15, -0.12)	-0.12 (-0.14, -0.10)
HbA1c, %)	-0.02 (-0.03, -0.01)	-0.02 (-0.03, -0.01)
Fasting plasma glucose, mg/dL	-0.44 (-0.74, -0.15)	-0.52 (-0.83, -0.22)
**Prevalent conditions†**		
Metabolic syndrome §	0.85 (0.82, 0.88)	0.83 (0.80, 0.86)
Diabetes ‖	0.96 (0.91, 1.01)	0.94 (0.89, 0.99)
Cardiovascular disease ¶	0.92 (0.88, 0.96)	0.91 (0.87, 0.95)
Cancer	0.95 (0.91, 0.99)	0.94 (0.90, 0.98)
Lung disease	0.92 (0.88, 0.96)	0.91 (0.87, 0.95)
Optimal cardiometabolic health €	1.24 (1.16, 1.32)	1.26 (1.18, 1.35)

* Individual Food Compass Score (i.FCS) calculated as the energy-weighted mean of FCS of all foods consumed, as reported in 24 hour dietary recall, ranging from 1 to 100.
† Survey-weighted, multivariable-adjusted linear regression (or logistic regression) models incorporated NHANES dietary sample weights to account for the complex survey design and response rates and provide nationally representative effect estimates (or odds ratios) and associated 95% confidence interval for the non-institutionalized U.S. population. All outcomes are reported in their units. All models adjusted for: age (years), age^2 (years), sex, race/ethnicity (Mexican-American, other Hispanic, non-Hispanic White, non-Hispanic Black, Asian/other race), education level (<HS graduate, HS graduate, some college or associates degree, ≥college graduate), income (poverty: income ratio), smoking status (non-smoker, former smoker, current smoker); total physical activity (MET-hours/week), alcohol use (%energy), and self-reported diabetes (yes, no).
‡ Additional covariates tested in sensitivity analyses included total energy (kcal, continuous), and survey cycle (categorical)
§ Defined according to AHA/NHLBI as the presence of three or more of: HDL-C < 40 mg/dL (men)/<50 mg/dL (women) or on drug treatment for low HDL-C; triglycerides ≥150 mg/dL or on drug treatment for elevated triglycerides; blood pressure ≥130 SBP or ≥85 DBP or on antihypertensive drug treatment with a history of hypertension; WC ≥102 cm (men)/ ≥88 cm (women); and fasting plasma glucose ≥100 mg/dL or on drug treatment for elevated glucose [1]
‖ Defined based on presence of at least one of : HbA1c level > 6.5%, fasting plasma glucose > 126 mg/dL, or use of diabetes medications.
¶ Defined based on the presence of both (1) self-reported coronary heart disease, heart failure, stroke, myocardial infarction, or angina and also (2) cardiovascular medication use including use of angina, hypertension, or lipid medications.

€ Defined based on optimal levels for each of the following: adiposity (BMI<25 kg/m^2 and WC ≤88 cm (women)/WC ≤102 cm (men)); blood glucose (FPG <100 mg/dL and HbA1c <5.7% and not taking diabetes medication); blood lipids (TC:HDL <3.5:1 and not taking lipid lowering medication); blood pressure (SBP < 120 mmHg, DBP < 80 mmHg and not taking blood-pressure lowering medication); and history of CVD (no self-reported CHD, heart failure, myocardial infarction, stroke, or angina).

BMI, body mass index; CI, confidence interval; CVD, cardiovascular disease; HbA1c, Hemoglobin A1c; HDL-C, high-density lipoprotein cholesterol; i.FCS, individual, dietary Food Compass Score; LDL-C, low density lipoprotein cholesterol; TC:HDL, total cholesterol to HDL-cholesterol ratio

Table S8. Multivariable adjusted, survey-weighted prospective analysis of individual Food Compass Score and the 9 components of the individual Food Compass Score with all-cause and cause-specific mortality among U.S. adults, 1999-2018

		Mortality (Hazard Ratio [95% CI])*		
		All Cause	CMD-specific†	Cancer-specific †
		N = 7481 deaths	*N = 2619 deaths*	*N = 1691 deaths*
**i.FCS‡**	model 1 §	0.93 (0.89, 0.96)	0.95 (0.89, 1.02)	0.92 (0.85, 1.00)
	model 2 §‖	0.93 (0.90, 0.97)	0.97 (0.90, 1.03)	0.93 (0.85, 1.01)
**i.Domain Scores‡**				
i.Nutrient Ratios	model1	0.92 (0.89, 0.96)	0.97 (0.9, 1.03)	0.90 (0.82, 0.98)
	model2	0.93 (0.89, 0.96)	0.97 (0.91, 1.03)	0.90 (0.83, 0.98)
i.Vitamins	model1	1.00 (0.96, 1.04)	1.01 (0.95, 1.07)	0.95 (0.88, 1.02)
	model2	1.00 (0.96, 1.04)	1.01 (0.95, 1.06)	0.96 (0.89, 1.02)
i.Minerals	model1	1.00 (0.95, 1.04)	1.00 (0.94, 1.07)	0.97 (0.90, 1.04)
	model2	1.00 (0.96, 1.05)	1.01 (0.95, 1.08)	0.97 (0.91, 1.04)
i.Food Ingredients	model1	0.95 (0.91, 0.99)	0.94 (0.88, 1.00)	0.98 (0.90, 1.06)
	model2	0.96 (0.92, 0.99)	0.96 (0.90, 1.02)	0.97 (0.90, 1.06)
i.Additives	model1	0.95 (0.92, 0.98)	0.98 (0.93, 1.05)	0.92 (0.86, 1.00)
	model2	0.95 (0.92, 0.98)	0.98 (0.93, 1.04)	0.94 (0.87, 1.01)
i.Processing	model1	0.93 (0.89, 0.96)	0.95 (0.90, 1.01)	0.95 (0.87, 1.04)
	model2	0.93 (0.90, 0.97)	0.97 (0.91, 1.03)	0.96 (0.88, 1.05)
i.Fiber and Protein	model1	0.93 (0.90, 0.96)	0.96 (0.91, 1.02)	0.94 (0.87, 1.02)
	model2	0.93 (0.90, 0.96)	0.96 (0.91, 1.02)	0.95 (0.89, 1.03)
i.Specific Lipids	model1	0.96 (0.92, 1.00)	0.97 (0.91, 1.03)	0.95 (0.87, 1.03)
	model2	0.96 (0.92, 1.00)	0.98 (0.92, 1.04)	0.94 (0.87, 1.02)
i.Phytochemicals	model1	0.98 (0.95, 1.02)	1.00 (0.95, 1.05)	0.97 (0.90, 1.05)
	model2	0.99 (0.96, 1.02)	0.99 (0.95, 1.05)	0.97 (0.91, 1.05)

* Survey-weighted Cox proportional hazard models incorporated NHANES dietary sample weights to account for the complex survey design and response rates and provide nationally representative estimates the hazard ratio and associated 95% confidence interval for the non-institutionalized U.S. population. Study time used as time variable in analysis (where baseline data collection: t = 0 yr).
† Cardiometabolic disease (CMD)-specific mortality is the sum of deaths from coronary heart disease, stroke, and diabetes. Cancer-specific mortality includes deaths from all cancer types.
‡ Individual Food Compass Score (i.FCS) calculated as the energy-weighted mean of FCS of all foods consumed, as reported in 24 hour dietary recall, ranging from 1 to 100. i.Domain Scores represent the nine component domains of the FCS at the individual level. For each i.Domain Score, the energy-weighted mean for all reported foods consumed was calculated, as with i.FCS. Hazard ratios reflect a 1 standard deviation increase in respective score.
§ adjusted for age (years), age^2 (years), sex, race/ethnicity (Mexican-American, other Hispanic, non-Hispanic White, non-Hispanic Black, Asian/other race), education level (<HS graduate, HS graduate, some college or associates degree, ≥ college graduate), income (poverty: income ratio), smoking status (non-smoker, former smoker, current smoker); total physical activity (MET-hours/week), alcohol use (%energy), and self-reported diabetes (yes, no).
‖ model 2 was further adjusted for HbA1c (%), systolic blood pressure (mm Hg), diastolic blood pressure (mm Hg), LDL-C (mg/dL), HDL-C (mg/dL), triglycerides (mg/dL), BMI (kg/m^2), and the following self-reported health conditions and medication usage: cardiovascular disease, cancer, lung disease, angina, diabetes medication, cholesterol medication, and angina medication.

CI, confidence interval; CMD, cardiometabolic disease; i.FCS, individual, dietary Food Compass Score

Table S9. Sensitivity analysis of the multi-variable adjusted, survey-weighted prospective association between individual Food Compass Score and all-cause and cause-specific mortality among U.S. adults, 1999-2018, censoring at 85+ years and incorporating additional covariates

	Hazard Ratio (95% CI) †		
**Mortality**	**primary analysis**	**censoring 85+ years ‡**	**Additional covariates (total energy + survey cycle) §**
All-cause	0.93 (0.89, 0.96)	0.91 (0.87, 0.95)	0.92 (0.89, 0.96)
Cardiometabolic disease-specific	0.95 (0.89, 1.02)	0.92 (0.85, 1.00)	0.95 (0.89, 1.02)
Cancer-specific	0.92 (0.85, 1.00)	0.91 (0.83, 1.00)	0.92 (0.84, 1.00)

* Individual Food Compass Score (i.FCS) calculated as the energy-weighted mean of FCS of all foods consumed, as reported in 24 hour dietary recall, ranging from 1 to 100. i.FCS was further standardized by dividing by the distribution's standard deviation (i.e. ~10 points).

† Survey-weighted Cox proportional hazard models incorporated NHANES dietary sample weights to account for the complex survey design and response rates and provide nationally representative estimates the hazard ratio and associated 95% confidence interval for the non-institutionalized U.S. population. Study time used as time variable in analysis (where baseline data collection: t = 0 yr). All models adjusted for age (years), age^2 (years), sex, race/ethnicity (Mexican-American, other Hispanic, non-Hispanic White, non-Hispanic Black, Asian/other race), education level (<HS graduate, HS graduate, some college or associates degree, ≥ college graduate), income (poverty: income ratio), smoking status (non-smoker, former smoker, current smoker); total physical activity (MET-hours/week), alcohol use (%energy), and self-reported diabetes (yes, no).

‡ All respondents deceased at age 85+ years censored (e.g., removed as events) in sensitivity analyses, with model otherwise identical to primary analysis.

§ Additional covariates tested included total energy intake (kcal) and survey cycle.

CI, Confidence Interval

Table S10. Multivariable adjusted, survey-weighted prospective association between individual Food Compass Score (i.FCS) and all-cause mortality, stratified by key sociodemographic factors, among U.S. adults, 1990-2018

			i.FCS, per SD (10.9 points) *
**Sociodemographic factor**	**N**	**Events (No. of deaths)**	**HR (95% CI)†**
**Sex**			
Male	22996	4158	0.93 (0.88, 0.98)
Female	24992	3323	0.93 (0.88, 0.98)
**Age category, years**			
20-64	36097	2222	0.90 (0.84, 0.96)
≥65	11839	5259	0.94 (0.89, 0.98)
**Race/ethnicity‡**			
Hispanic American	12311	1270	0.88 (0.78, 0.98)
Non-Hispanic White	21569	4512	0.93 (0.89, 0.98)
Non-Hispanic Black	9938	1462	0.95 (0.89, 1.02)
**Education level**			
≤HS graduate	23882	4841	0.94 (0.89, 0.99)
>HS graduate	24036	2651	0.90 (0.85, 0.95)
**Income, PIR§**			
Low income (PIR ≤2.16)	23832	4370	0.91 (0.86, 0.96)
High income (PIR >2.16)	24086	3111	0.93 (0.89, 0.98)
**BMI, kg/m²**			
Normal or underweight (<25)	14124	2333	0.89 (0.84, 0.94)
Overweight (25-29)	17569	2636	0.94 (0.89, 1.00)
Obese (BMI ≥ 30)	16225	2512	0.94 (0.88, 1.01)
**Energy reporting, TEI:BMR ‖**			
Under-report	12039	2105	0.94 (0.87, 1.01)
Acceptable	34568	4927	0.90 (0.87, 0.94)

* Individual Food Compass Score (i.FCS) calculated as the energy-weighted mean of FCS of all foods consumed, as reported in up to two 24 hour dietary recalls per person, with a potential range from 1 to 100.

† Survey-weighted, multivariable-adjusted Cox proportional hazard model incorporated NHANES dietary recall sample weights to account for the complex survey design and response rates and provide nationally representative effect estimates and associated 95% confidence interval for the non-institutionalized U.S. population. All models adjusted for: age (years), age² (years), sex, race/ethnicity (Mexican-American, other Hispanic, non-Hispanic White, non-Hispanic Black, Asian/other race), education level (<HS graduate, HS graduate, some college or associates degree, ≥ college graduate), income (poverty: income ratio) unless stratifying for that variable; and smoking status (non-smoker, former smoker, current smoker); total physical activity (MET-hours/week), alcohol use (%energy), and self-reported diabetes (yes, no).

‡ Mexican-American and Other Hispanic combined, and Asian/other excluded, from stratified analysis given small sample size and number of events.

§ Represents the ratio of family income to the federal poverty threshold, adjusted for household size. A higher ratio indicates a higher level of income. Individuals were grouped into low and high income, with the cut-point drawn at the median PIR (2.160).

‖ TEI:BMR, the ratio of average total energy intake (TEI, in kcal/day) to basal metabolic rate (BMR, kcal/day), was used to determine the plausibility of total energy reporting based on Goldberg cut-offs. BMR was estimated via the Schofield sex- and age-specific predictive equations based on weight and height. TEI was calculated based on the total number of recall days available for each respondent. For example, when two days of data were available, an arithmetic average TEI was calculated. Under-reporters, acceptable reporters and over-reporters were defined as having EI:BMR<0·96, 0·96–2·49 and >2·49 for 2-day dietary recall data, and <0·87, 0·87–2·75 and >2·75 for 1-day dietary recall data, respectively, as previously derived in NHANES.[5] Over-reporters were excluded from sub-group analyses given sample size and number of events.

AA, Associates Degree; BMI, body mass index; BMR, basal metabolic rate; CI, confidence interval; HS, high school; i.FCS, individual, dietary Food Compass Score; PIR, poverty: income ratio; SD, standard deviation; TEI, total energy intake

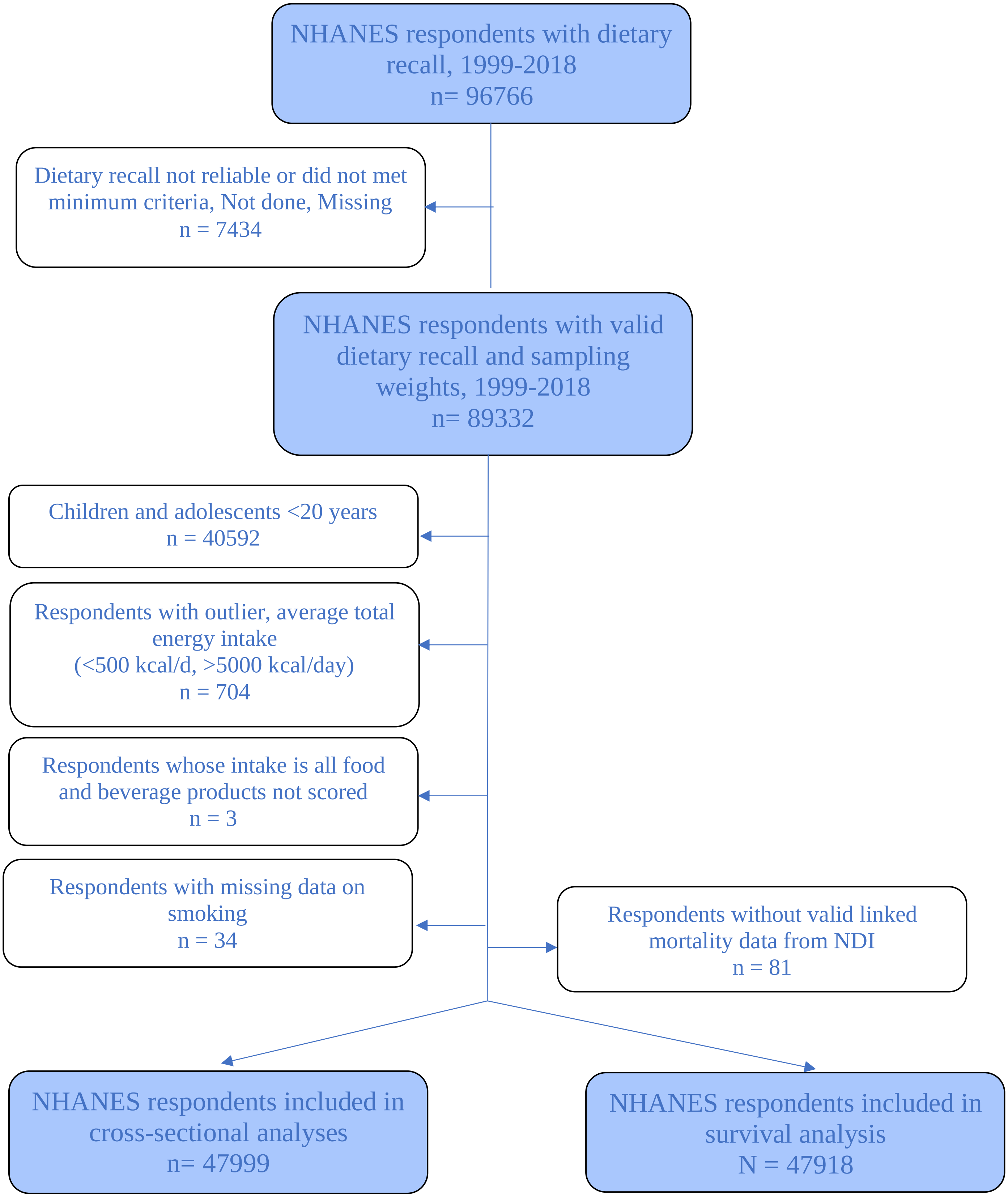

Figure S1. Flow diagram for inclusion of NHANES participants in cross-sectional and survival analysis. We excluded respondents without valid dietary recalls (n=7434), with extreme total energy intake (<500 or >5000 kcal/day; n=704), who only reported intake of alcoholic beverages (n=3), or with missing data on smoking status (n=34). For prospective mortality analyses, we further excluded respondents without valid linked mortality data (n=81).

Kcal, kilocalorie; NDI, National Death Index; NHANES, National Health and Nutrition Examination Survey

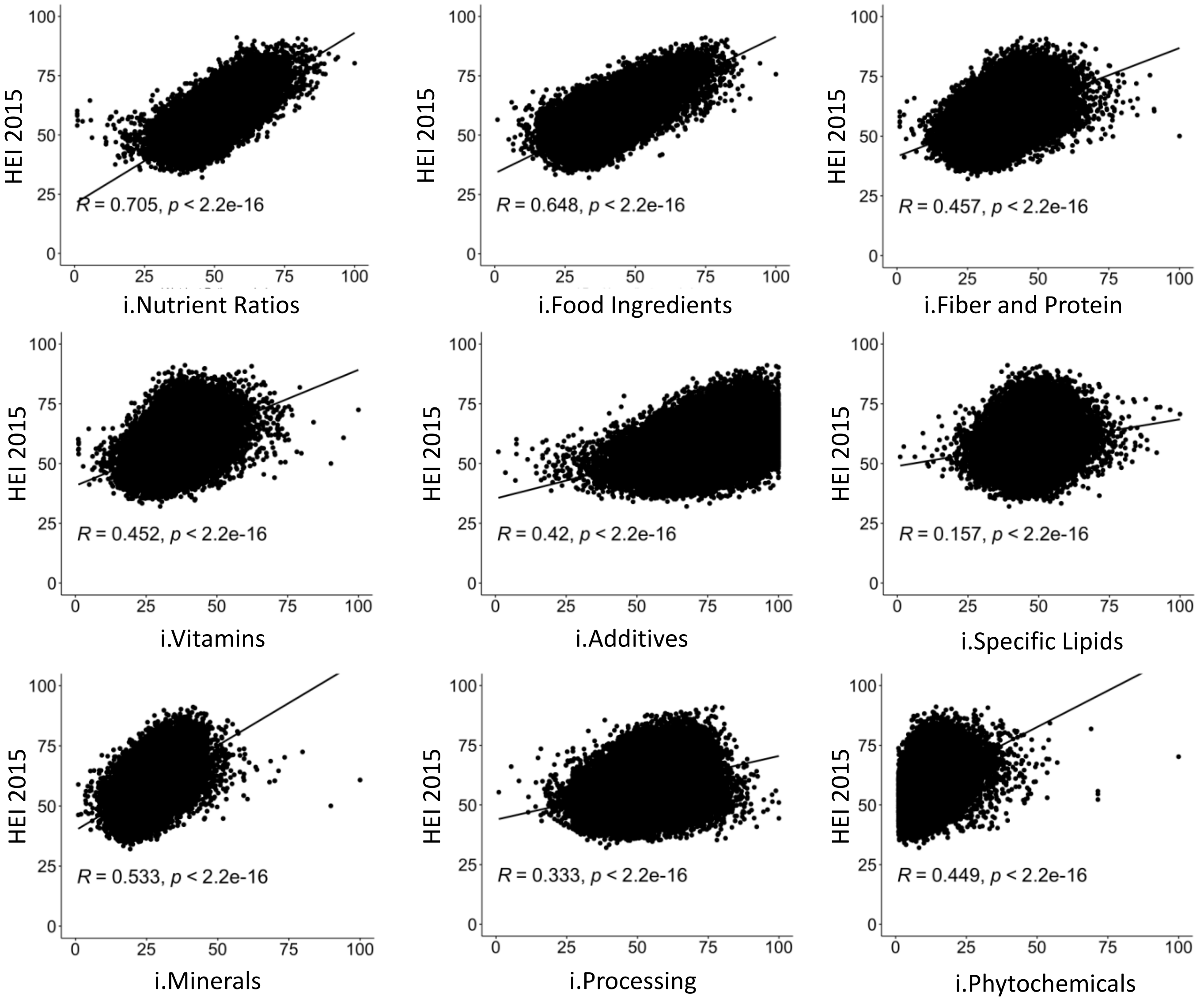

Figure S2. Relationship between Healthy Eating Index (HEI) 2015 and the 9 components of the individual Food Compass Score (i.FCS) among U.S. adults, 1999-2018. Component i.Domain Scores calculated by taking the energy-weighted mean of the respective score for all foods consumed by that individual reported in 24-hour dietary recall, as with i.FCS. Black dots represent each NHANES respondent; solid lines, the line of best fit between all NHANES respondents; and R values, the correlation and p-value(2-sided) for the Spearman correlation.

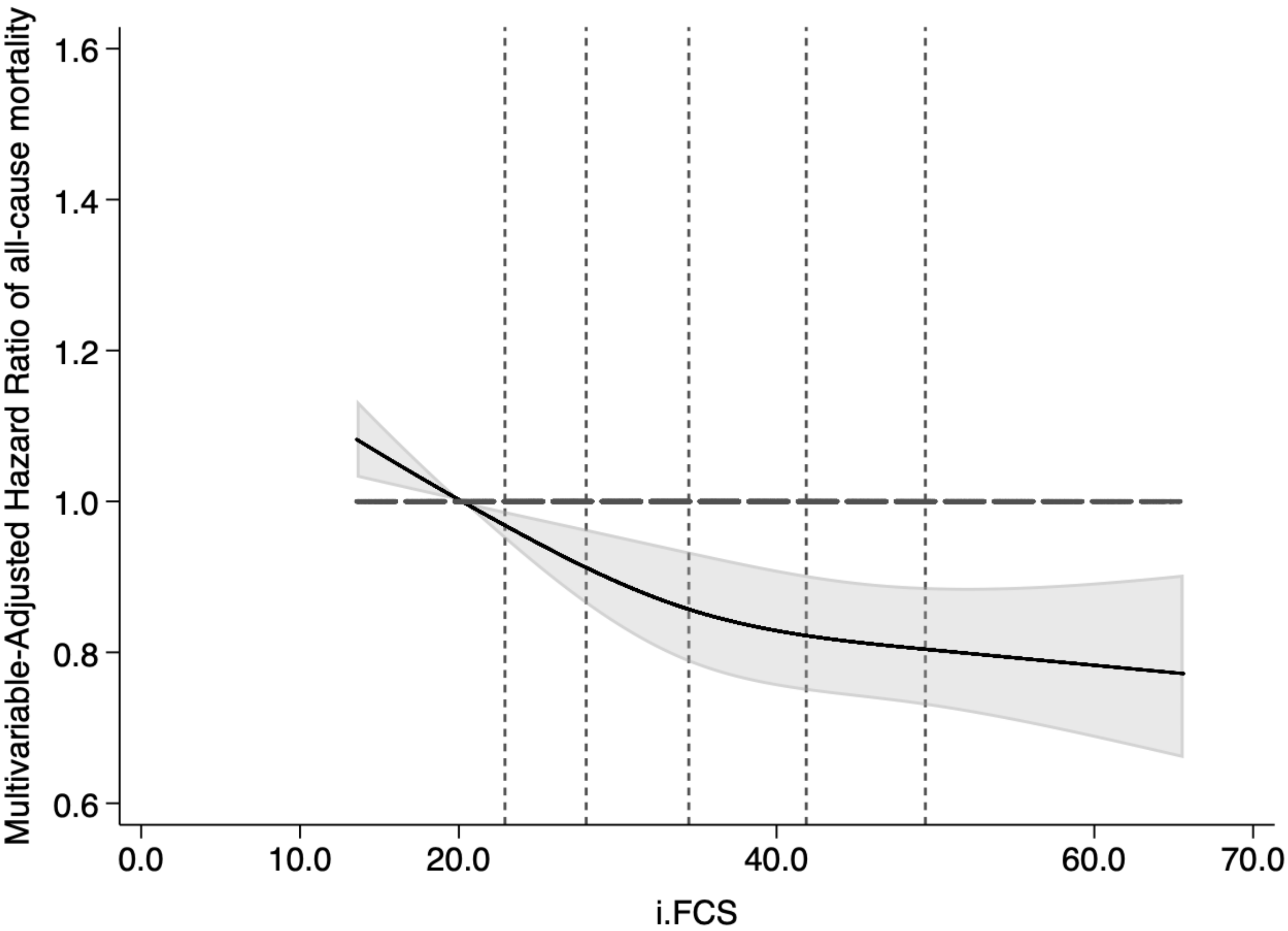

Figure S3. Semi-parametric, restricted cubic spline analysis testing non-linearity in the association between individual Food Compass Scores (i.FCS) and all-cause mortality among U.S. adults, 1999-2014. The i.FCS was calculated as the energy-weighted mean FCS of all foods and beverages consumed by each person, as reported in up to two 24 hour dietary recalls per person, and could range from 1 to 100. Risk was assessed using survey-weighted Cox proportional hazards, incorporating NHANES dietary sample weights to account for the complex survey design and response rates and provide nationally representative estimates. Covariates included age (years), age^2 (years), sex, race/ethnicity (Mexican-American, other Hispanic, non-Hispanic White, non-Hispanic Black, Asian/other race), education level (<HS graduate, HS graduate, some college or associates degree, ≥college graduate), income (poverty: income ratio), smoking status (non-smoker, former smoker, current smoker); total physical activity (MET-hours/week), alcohol use (%energy), and self-reported diabetes (yes, no). Analyses excluded the top and bottom 1% of i.FCS scores, with the 5th percentile of i.FCS as the reference value. An adjusted Wald test was used to assess statistically significant differences between the linear vs. restricted cubic spline models. The solid line represents the estimated hazard ratio; the shaded area, the 95% CI; and the dotted vertical lines, the 10th, 25th, 50th, 75th, and 90th percentile values for i.FCS.

i.FCS, individual Food Compass Score

Supplementary Note 1. Identification of complete and reliable dietary recall observations in NHANES, 1999-2018

We used all complete and reliable dietary records available in the NHANES 24 dietary assessment,[6] as determined by the National Center for Health statistics staff, averaging the two recalls for each individual when present to reduce the influence of day-to-day dietary variation.

Reliable 24-hour dietary recalls were defined as meeting the following criteria in 1990-2000 survey cycle:

- Less than 25% foods with missing descriptive information [e.g., caffeinated or decaffeinated, preparation methods, or brand names]
- Less than 15% foods with missing amounts, and any meal reported must have at least one known food;

And the following updated criteria for the 2001-2 to 217-18 survey cycles:

- First four steps of the 5-step USDA automated multiple-pass method (AMPM) completed and food/beverages consumed for each reported eating occasion[6]

Supplementary Note 2. Missing attribute values in FNDDS 2001-2018 for nutrient profiling

Attributes required for Food Compass scoring that were missing across the entire FNDDS database from 2001-2018 were excluded from our analyses, as done previously,[7] including: iodine; artificial sweeteners, flavors or colors; partially hydrogenated oils; interesterified or hydrogenated oils; high-fructose corn syrup; monosodium glutamate (MSG); and trans fats.

For the total flavonoids, vitamin D and choline attributes, values were missing for food products only in particular survey cycles. We used two sequential methodologies for handling missing values for these remaining, partially missing Food Compass attributes. First, for specific products (unique food codes) with attribute data available in some edition of the FNDDS database, the attribute value per standard quantity (e.g. per 100 kcal) in the most proximal year available was carried backwards to any preceding year with that attribute missing, or carried forwards to any following year with the attribute missing.

Second, for specific products without attribute data available in any of the FNDDS database editions, we used predictive mean matching (PMM) imputation to address the remaining missing values. PMM is a semi-parametric imputation approach, whereby for each missing value, it fills in a value randomly from among the observed donor values from an observation whose regression-predicted values are closest to the regression-predicted value for the missing value from the simulated model.[8,9] Such imputation techniques ensure that imputed values are plausible, and is particularly useful when the normality assumption required for standard regression methods are violated. For total flavonoids, the predictors used in the imputation model included: all 10 food-based ingredient FPED values, and indicator variables for each of the 152 What We Eat in America (WWEIA) categories that all reported food and beverage items are categorized into – the most granular categorization available. For vitamin D and choline, predictors included: all 10 food-based ingredient FPED values, all macronutrient and micronutrient values from FNDDS, and the 152 WWEIA categories as indicator variables.

Table S11. Missing values for total flavonoids, vitamin D, and choline in FNDDS 2001-2018

Attribute	Observations with missing values in FNDDS 2001-2018 (%)	Unique food codes with missing values (%)
Total flavonoids	8640 (13.9%)	3390 (33.3%)
Vitamin D	458 (0.7%)	200 (2.0%)
Choline	420 (0.7%)	187 (1.8%)

In addition, FNDDS does not provide a database corresponding to the 1999-2000 NHANES survey cycle. For each product reported by NHANES respondents in 1999-2000, we generated a “mock” FNDDS 1999-2000 database, by copying over the nutritional data for each product food code from the most proximal available year. After matching products reported in NHANES 1999-2000 to later FNDDS databases, 115 unique products remained without an exact food code match. We used the product description for these remaining 115 products to assign a food code from the earliest edition of the FNDDS database

Supplementary Note 3. Modifications to the Food Compass algorithm

Since the publication of the original Food Compass paper,[7] a few relevant modifications to the Food Compass algorithm were identified and implemented. These included:

1. **A more graded approach to scoring fermentation**

Food products were scored for fermentation using percentage of energy from yogurt or cheese ingredients as a proxy, with 50% energy from yogurt or cheese assigned the target high score (10) and linear scaling down to 0% energy from yogurt or cheese assigned the target low score (0). Cheese and yogurt food pattern equivalents data from the Food Pattern Equivalents Database (FPED) was converted from cup-equivalents per 100 g to %kcal using the following conversion factors, based on the average kcal per 100 grams of ingredient:

** 245 g per 1 cup-equivalent of yogurt; 82.1 kcal per 100g yogurt **

** 113 g per 1 cup-equivalent of cheese; 271.4 kcal/100g cheese **

In addition, products were identified as fermented and assigned the target high score (10) if their description included at least one of the following keywords: ferment, culture, sour dough/ sourdough, sauerkraut, kimchi, natto, miso, kefir, kombucha, injera, dosa.

Previously, fermentation was a dichotomous variable, with any product containing ≥50% energy from yogurt or cheese, or identified by keyword search, classified as fermented (10); and all other products classified as unfermented (0)

2. **A more graded approach to scoring nitrites**

Food products were scored for nitrites using percentage of energy from cured or processed meat ingredients as a proxy, with 25% energy from cured or processed meat assigned the target low score (-10) and linear scaling up to 0% energy from cured or processed meat assigned the target high score (0). Cured and processed meat food pattern equivalents data from FPED was converted from ounce-equivalents per 100 g to %kcal using the following conversion factors, based on the average calorie per 100 grams of cured or processed meats:

** 28.35 g per 1 oz-equivalent cured meat; 270.5 kcal per 100g cured meat **

Previously, nitrites was a dichotomous variable, with any product containing ≥25% energy from cured or processed meats classified as containing nitrites (-10); and all other products classified as nitrite-free (0).

3. **Alternative data source and scoring for NOVA processing classification**

The NOVA processing classification was scored as -10, -5, -2.5, and 0 for the 4 categories of ultra-processed, processed, culinary ingredients, and un-processed/minimally foods, respectively. For mixed dishes (about 2/3 of all products), NOVA classification was calculated based on an energy-weighted score of the constituent ingredients' NOVA classification, leading to non-integer NOVA classification values. As such, attribute scores for NOVA classification were linearly scaled between the integer NOVA classes 1-4.

Previously, NOVA processing classification was obtained from a machine learning-based algorithm that predicts the degree of processing of any food or beverage product.[10] Given the emerging nature of that work, we opted to utilized the manual NOVA classification scores calculated by the Monteiro research

group.[11] We believe these manual scores better aligns with epidemiological analyses of ultra-processed foods and health outcomes in the literature.[12]

4. Addition of fruit juice and vegetable juice as Food Ingredient attributes

Fruit juice and vegetable juice were added as Food Ingredient attributes, with maximum high scores of 5 (all other healthful attributes had maximum high score of 10), given the equivocal nature of their association with health outcomes. Target high scores for both were based on the 95th percentile of relevant foods assessed, as done for other Food Ingredient attributes. As with the original algorithm, the attribute scores in this domain were summed (not averaged), because contents of each ingredient are mutually interdependent.

5. Refinement of target low and high scores for Food Ingredients attributes

To determine the target low and high scores for each Food Ingredient attribute, the 95th percentile for each food ingredient was calculated as a subset of relevant foods assessed from FNDDS 2001-2018 (i.e., yogurt for the food-based yogurt attribute). Separate 95th percentile values were calculated and used for dried vs. non-dried fruits and dried vs. non-dried, non-starchy vegetables given their different water weights. The table below provides the target low and high scores for each food ingredient attribute from the modified vs. original algorithm, as well as the relevant foods assessed (n) for each food ingredient attribute in FNDDS 2001-2018.

Table S12. Comparison of modified vs. original Food Compass algorithm target low and high scores for food ingredients domain attributes

Food Ingredient Attributes (per 100 kcal)	Attribute Points	modified algorithm, FNDDS 2001-2018			*original algorithm, FNDDS 2015-16*	
		Target for low score	Target for high score	relevant foods assessed	*Target for low score*	*Target for high score*
Fruits	0 to 10	0	1.75	*non-dried fruits in fruit category (n = 1665)*	*0*	*1.67 cups*
Fruits, dried	0 to 10	0	0.75	*dried fruit (n= 324)*	*0*	*0.7 cups*
Fruit juice	0 to 5	0	1.08	*fruit juice (n=333)*	-	-
Vegetables, non-starchy	0 to 10	0	4.77	*non-starchy, non-dried vegetables in vegetable category (n = 6003)*	*0*	*3.29 cups*
Vegetables, non-starchy, dried	0 to 10	0	4.18	*non-starchy, dried vegetables (n=53)*	*0*	*1.55*
Vegetable juice	0 to 5	0	2.41	*vegetable juice (n=91)*	-	-
Beans and legumes	0 to 10	0	0.5	*legumes (n=1057)*	*0*	*0.44 cups*
Whole grains	0 to 10	0	1.12	*grains (n = 6195)*	*0*	*1.01 oz*
Nuts and seeds	0 to 10	0	1.35	*nuts and seeds (n=601)*	*0*	*1.22 oz*
Seafood	0 to 10	0	3.86	*seafood (n =2289)*	*0*	*3.89 oz*
Yogurt	0 to 10	0	0.81	*yogurt (n = 221)*	*0*	*0.55 cups*
Plant Oils	0 to 10	0	11.31	*plant oils (n=780)*	*0*	*11.31 g*
Refined grains	-10 to 0	1.45	0	*grains (n= 6195)*	*1.36 oz*	*0*

Red or processed meat	-10 to 0	2.69	0	*beef, lamb, game, and cured meat (n=2474)*	*2.25 oz*	*0*

Previously, the 95th percentile values for target low and high scores were calculated based on a broader range of relevant foods (i.e., dairy for the food-based yogurt attribute), and only for products within the FNDDS 2015-16.

6. Alternative imputation methods for missing attribute values

For total flavonoids, vitamin D and choline attributes, values were missing for food products only in particular survey cycles. For specific products without attribute data available in any of the FNDDS database editions, we used predictive mean matching (PMM) imputation to address the remaining missing values. See Text S1 for missing value statistics and more detailed imputation methods.

Previously, for products without total flavonoids, vitamin D or choline values, the mean value of the What We Eat In America (WWEIA) category that that product was categorized into was used to fill in the respective missing value.

7. Updated truncation limits for scaling FCS from 1 to 100.

The truncation and subsequent scaling of FCS scores to 100 in this analysis was based on all 58,622 scored food and beverage items from all versions of FNDDS 2001-2018. The distribution was first truncated at the 5th and 95th percentiles (-10.4, 28.4). The final Food Compass Score (FCS) was then scaled across all food and beverage items to range from 1 (least healthful) to 100 (most healthful) using the equation: FCS = [100 – (((max score – original score)/score range))*99)], or [100 – (((28.4 – original score)/38.8))*99)].

*Previously, the FCS scores were truncated at the 5th and 95th percentile of the 8,032 foods and beverages in FNDDS 2015-16 (-10.7, 26.1). As such, the final Food Compass Score (FCS) was then scaled across all 8032 food and beverage items using the equation: FCS = [100 – (((26.1– original score)/36.7))*99)].*

Supplementary Note 4. Development of total physical activity (MET-hours/week) variable in NHANES, 1999-2018

Total physical activity, assessed continuously as Metabolic Equivalent of Task (MET)-hours per week, was used as a covariate in all multivariable adjusted cross-sectional and longitudinal analyses. Generally, Standardized MET-scores -the ratio of the metabolic rate to the resting metabolic rate for key activities – were multiplied by the respondent's self-reported time (in hours per week) spent on physical activity and summed across all activities. However, methodological differences in how physical activity was assessed in NHANES from 1999-2006 and from 2007-2018 preclude the use of a consistent variable for total physical activity across survey cycles. As such, we used a total physical activity variable (MET-hours/week) evaluated from 2007-2018, a leisure time physical activity (MET-hours/week) for all survey cycles, and all other demographic and health data to impute total physical activity (MET-hours/week) for 1999-2006 survey cycles using predictive mean matching imputation.

For example, we calculated leisure time physical activity (LTPA) as follows:

<u>1999-2006 survey cycles:</u>
Two datasets were available across these survey cycles based on a general physical activity questionnaire and individual activity questionnaire. For calculating LTPA, only the individual activity questionnaire was used. For each respondent, we calculated MET-hours/week by multiplying self-reported frequency per week by self-reported duration in hours and self-reported level of intensity (moderate: MET = 4.0; vigorous: MET = 8.0), and summed across all individual activities. For individuals that did not report on any of the individual activities, their MET-hours/week value was considered 0.

<u>2007-2018 survey cycles:</u>
One dataset was available across these survey cycles where respondents reported on physical activity, disaggregated by "recreational" versus "work-related", and by "moderate" versus "vigorous". For each respondent, we calculated MET-hours/week by multiplying self-reported frequency per week by self-reported duration in hours and self-reported level of intensity (moderate: MET = 4.0; vigorous: MET = 8.0), and summed the "vigorous" and "moderate" recreational activities only. For individuals that did not report on recreational activities, their MET-hours/week value was considered.

To impute total physical activity for the 1999-2006 survey cycles, we used the following predictors: all sociodemographic (i.e., age, sex, race/ethnicity, education level, etc.), biomarker (i.e., BMI, systolic blood pressure, fasting plasma glucose, etc.) and health conditions (i.e., stroke, emphysema, etc.), leisure time physical activity (MET-hours/week), and total physical activity (calculated from 2007-2018 survey cycles only). Predictive mean matching (see Text S1 for more details) imputation was used to address missing values.

Survey-weighted means for total physical activity (imputed and pre-imputation) and leisure time physical activity by survey cycle, overall and by BMI category, are reported below:

Table S13. Survey-weighted mean total physical activity (MET-hours/week) calculated based on imputation among U.S. adults, 1999 -2018

Subgroup	1999-2000	2001-2002	2003-2004	2005-2006	2007-2008	2009-2010	2011-2012	2013-2014	2015-2016	2017-2018	p-trend
**all**	65 (60-70)	68 (62-74)	60 (55-66)	65 (60-71)	68 (63-73)	56 (51-61)	56 (51-60)	55 (51-60)	66 (60-71)	77 (70-84)	0.45
BMI<25	72 (62-83)	69 (61-77)	66 (57-74)	75 (65-85)	70 (61-80)	59 (52-65)	62 (58-66)	59 (52-66)	73 (64-81)	83 (71-95)	0.78
BMI 25-30	67 (62-72)	69 (63-74)	56 (47-66)	63 (58-68)	67 (61-74)	61 (55-67)	55 (47-62)	62 (54-69)	69 (58-81)	75 (66-85)	0.3
BMI 30+	54 (47-61)	66 (55-78)	58 (49-68)	58 (50-66)	65 (57-73)	49 (42-56)	51 (42-59)	48 (44-51)	58 (53-63)	75 (68-81)	0.27

Table S14. Survey-weighted mean total physical activity (MET-hours/week) without imputation among U.S. adults, 1999 -2018

Subgroup	1999-2000	2001-2002	2003-2004	2005-2006	2007-2008	2009-2010	2011-2012	2013-2014	2015-2016	2017-2018	p-trend
overall	23 (21-25)	22 (20-24)	24 (21-27)	26 (23-29)	68 (63-73)	56 (51-61)	56 (51-60)	55 (51-60)	66 (60-71)	77 (70-84)	<0.001
BMI<25	22 (19-26)	24 (21-27)	23 (20-25)	29 (25-33)	71 (61-80)	59 (52-65)	62 (58-66)	59 (52-66)	73 (64-82)	83 (71-95)	<0.001
BMI 25-30	27 (23-31)	23 (20-26)	27 (23-30)	25 (22-28)	67 (61-74)	61 (55-67)	55 (47-62)	62 (54-69)	69 (58-81)	75 (66-85)	<0.001
BMI 30+	19 (17-22)	20 (17-22)	24 (18-30)	25 (21-28)	65 (57-73)	49 (42-56)	51 (42-59)	48 (44-51)	58 (53-63)	75 (68-81)	<0.001

Table S15. Survey-weighted mean leisure-time physical activity (MET-hours/week) among U.S. adults, 1999 -2018

Subgroup	1999-2000	2001-2002	2003-2004	2005-2006	2007-2008	2009-2010	2011-2012	2013-2014	2015-2016	2017-2018	p-trend
overall	19 (16-22)	23 (19-28)	16 (15-18)	20 (18-21)	14 (12-16)	14 (12-16)	16 (13-18)	15 (13-16)	16 (14-17)	16 (14-17)	<0.001
BMI<25	21 (17-25)	25 (20-30)	19 (15-22)	24 (21-28)	17 (15-20)	17 (14-20)	21 (17-26)	19 (17-22)	21 (17-24)	18 (15-21)	0.06
BMI 25-30	21 (17-25)	25 (19-31)	16 (13-18)	19 (18-21)	15 (12-18)	14 (12-16)	16 (13-19)	16 (13-18)	18 (15-21)	17 (15-19)	0.005
BMI 30+	15 (13-17)	19 (14-25)	14 (12-16)	15 (14-17)	10 (8-11)	11 (9-12)	10 (8-12)	10 (9-12)	11 (10-12)	14 (12-16)	0.001

Body mass index, BMI; Metabolic equivalents of Task, MET

Supplementary Note 5. Missing values for clinical risk factors and health conditions in NHANES, 1999-2018.

NHANES uses trained personnel and standardized methods including in-person physical examinations to collect information on clinical risk factors and health conditions in their recurrent cross-sectional, nationally representative samples.

Measures for several clinical risk factors – including fasting plasma glucose, triglycerides and LDL-cholesterol – were only collected on a subsample of NHANES respondents who were examined in the morning session only and who fasted at least 8.5 hours, but less than 24 hours. There was also missing values at random for other clinical risk factors, self-reported health conditions and medication usage. The table below provided the extent of missing values (n (%)) within the repeat cross-sectional population assessed in this analysis (n = 47999):

Table S16. Missing values for clinical risk factors and health conditions in NHANES, 1999-2018

**clinical risk factors**	**missing (n (%))**
total cholesterol, mg/dL	2481 (5.2)
HDL-C, mg/dL	2483 (5.2)
LDL-C, mg/dL	26815 (55.9)
triglycerides, mg/dL	26030 (54.2)
waist circumference, cm	1795 (3.7)
BMI, kg/m^2	740 (1.5)
HbA1c, %	1968 (4.1)
fasting plasma glucose, mg/dL	27334 (57.0)
systolic blood pressure, mm Hg	1476 (3.1)
diastolic blood pressure, mm Hg	1476 (3.1)
**self-reported health conditions and medication usage**	
self-reported diabetes	25 (0.1)
self-reported congestive heart failure	147 (0.3)
self reported coronary heart disease	211 (0.4)
self-reported myocardial infarction	77 (0.2)
self-reported stroke	59 (0.1)
self-reported cancer	42 (0.1)
self-reported emphysema	58 (0.1)
self-reported bronchitis	96 (0.2)
self-reported asthma	44 (0.1)
self-reported COPD	33408 (69.6)
self-reported angina	19681 (41)
diabetes medication usage	479 (1)
cholesterol medication usage	481 (1)
angina medication usage	573 (1.2)
hypertension mediation usage	435 (0.9)

To address missing values, we used predictive mean matching (PMM) –a semi-parametric imputation approach whereby for each missing value, it fills in a value randomly from among the observed donor values from an observation whose regression-predicted values are closest to the regression-predicted value for the missing value from the simulated model, as previously described[8,9] Thus, there were no missing values in either the present cross-sectional or survival analysis.

Supplementary References

1. Grundy, SM, Cleeman, JI, Daniels, SR, et al. Diagnosis and Management of the Metabolic Syndrome. Circulation. 2005; 112(17): 2735-2752. doi:doi:10.1161/CIRCULATIONAHA.105.169404
2. American Diabetes Association. (2021). Understanding A1c: Diagnosis. Available at: https://www.diabetes.org/a1c/diagnosis Accessed June 16, 2021
3. Calling, S, Johansson, S-E, Wolff, M, Sundquist, J, & Sundquist, K. The ratio of total cholesterol to high density lipoprotein cholesterol and myocardial infarction in Women's health in the Lund area (WHILA): a 17-year follow-up cohort study. BMC Cardiovascular Disorders. 2019; 19(1): 239. doi:10.1186/s12872-019-1228-7
4. Unger, T, Borghi, C, Charchar, F, et al. 2020 International Society of Hypertension Global Hypertension Practice Guidelines. Hypertension. 2020; 75(6): 1334-1357. doi:doi:10.1161/HYPERTENSIONAHA.120.15026
5. Murakami, K, & Livingstone, MB. Prevalence and characteristics of misreporting of energy intake in US adults: NHANES 2003-2012. Br J Nutr. 2015; 114(8): 1294-1303. doi:10.1017/s0007114515002706
6. Centers for Disease Control and Prevention. (2013). National Health and Nutrition Examination Survey. Available at: http://www.cdc.gov/nchs/nhanes.htm Accessed April 15, 2020
7. Mozaffarian, D, El-Abbadi, NH, O'Hearn, M, et al. Food Compass is a nutrient profiling system using expanded characteristics for assessing healthfulness of foods. Nature Food. 2021; 2(10): 809-818. doi:10.1038/s43016-021-00381-y
8. Heitjan, DF, & Rubin, DB. Inference from Coarse Data Via Multiple Imputation with Application to Age Heaping. Journal of the American Statistical Association. 1990; 85(410): 304-314. doi:10.2307/2289765
9. Schenker, N, & Taylor, JMG. Partially parametric techniques for multiple imputation. Computational Statistics & Data Analysis. 1996; 22(4): 425-446. doi:https://doi.org/10.1016/0167-9473(95)00057-7
10. Menichetti, G, Ravandi, B, Mozaffarian, D, & Barabási, A-L. Machine Learning Prediction of Food Processing. medRxiv. 2022: 2021.2005.2022.21257615. doi:10.1101/2021.05.22.21257615
11. Monteiro, CA, Cannon, G, Levy, RB, et al. Ultra-processed foods: what they are and how to identify them. Public Health Nutrition. 2019; 22(5): 936-941. doi:10.1017/S1368980018003762
12. Askari, M, Heshmati, J, Shahinfar, H, Tripathi, N, & Daneshzad, E. Ultra-processed food and the risk of overweight and obesity: a systematic review and meta-analysis of observational studies. International Journal of Obesity. 2020; 44(10): 2080-2091. doi:10.1038/s41366-020-00650-z
