# Supplementary material for: Validation of Food Compass with a healthy diet, cardiometabolic health, and mortality among U.S. adults, 1999–2018
Source: Nat Commun. 2022 Nov 22;13:7066. doi: 10.1038/s41467-022-34195-8 (PMC9681774; doi:10.1038/s41467-022-34195-8)
Supplement: Supplementary file 3 — Reporting Summary [file 41467_2022_34195_MOESM3_ESM.pdf]

nature portfolio

Corresponding author(s): Meghan O'Hearn

Last updated by author(s): 10/12/2022

Reporting Summary

Nature Portfolio wishes to improve the reproducibility of the work that we publish. This form provides structure for consistency and transparency in reporting. For further information on Nature Portfolio policies, see our Editorial Policies and the Editorial Policy Checklist.

Statistics

For all statistical analyses, confirm that the following items are present in the figure legend, table legend, main text, or Methods section.

n/a	Confirmed	
[ ]	[x]	The exact sample size (*n*) for each experimental group/condition, given as a discrete number and unit of measurement
[ ]	[x]	A statement on whether measurements were taken from distinct samples or whether the same sample was measured repeatedly
[ ]	[x]	The statistical test(s) used AND whether they are one- or two-sided<br>*Only common tests should be described solely by name; describe more complex techniques in the Methods section.*
[ ]	[x]	A description of all covariates tested
[ ]	[x]	A description of any assumptions or corrections, such as tests of normality and adjustment for multiple comparisons
[ ]	[x]	A full description of the statistical parameters including central tendency (e.g. means) or other basic estimates (e.g. regression coefficient) AND variation (e.g. standard deviation) or associated estimates of uncertainty (e.g. confidence intervals)
[ ]	[x]	For null hypothesis testing, the test statistic (e.g. *F*, *t*, *r*) with confidence intervals, effect sizes, degrees of freedom and *P* value noted<br>*Give P values as exact values whenever suitable.*
[x]	[ ]	For Bayesian analysis, information on the choice of priors and Markov chain Monte Carlo settings
[x]	[ ]	For hierarchical and complex designs, identification of the appropriate level for tests and full reporting of outcomes
[x]	[ ]	Estimates of effect sizes (e.g. Cohen's *d*, Pearson's *r*), indicating how they were calculated

Our web collection on statistics for biologists contains articles on many of the points above.

Software and code

Policy information about availability of computer code

Data collection	All data was downloaded directly from sources specified in data availability statement. No software was used for data collection.
Data analysis	Custom code was developed using in R (Version 4.0.3) and Stata SE (Version 15.1) for data cleaning, adjustments, and merging, algorithm scoring of FCS for individual food and beverage items, i.FCS scoring of individuals, cross-sectional and survival analyses of i.FCS with health outcomes, and summary statistics, tables, and figures. Statistical significance was defined as two-tailed alpha=0.05. Tufts University is considering licensing of expertise, knowledge, and background work for potential commercial viability of Food Compass for private sector and non-profit applications, and thus the code is not publicly available. There are no intellectual property or patent protections associated with Food Compass. The detailed Food Compass algorithm and it scoring are specified in our earlier Nature Food Publication, as well as in Table S2 of the current supplementary materials, and can be reproduced freely by any individual or organization.

For manuscripts utilizing custom algorithms or software that are central to the research but not yet described in published literature, software must be made available to editors and reviewers. We strongly encourage code deposition in a community repository (e.g. GitHub). See the Nature Portfolio guidelines for submitting code & software for further information.

Data

Policy information about availability of data

All manuscripts must include a data availability statement. This statement should provide the following information, where applicable:
- Accession codes, unique identifiers, or web links for publicly available datasets
- A description of any restrictions on data availability
- For clinical datasets or third party data, please ensure that the statement adheres to our policy

The attribute and domain scoring algorithm used to generate the Food Compass is available in the Supplementary Information of the following publication: https://www.nature.com/articles/s43016-021-00381-y
All data used in this analysis is publicly available from the following USDA and CDC sources:
(1) Nutrient composition data for foods reported in NHANES dietary recalls [USDA Food and Nutrient Database for Dietary Studies 2001-2018, FNDDS: https://www.ars.usda.gov/northeast-area/beltsville-md-bhnrc/beltsville-human-nutrition-research-center/food-surveys-research-group/docs/fndds-download-databases/]
(2) Food ingredients data for foods reported in NHANES dietary recalls [USDA Food Pattern Equivalents Database, 2001-2018: https://www.ars.usda.gov/northeast-area/beltsville-md-bhnrc/beltsville-human-nutrition-research-center/food-surveys-research-group/docs/fped-overview/]
(3) Flavonoid data for select foods reported in NHANES dietary recalls [USDA Flavonoid Database, 2007-2010: https://data.nal.usda.gov/dataset/usda-database-flavonoid-content-selected-foods-release-32-november-2015
(4) National dietary recall, sociodemographic, physical activity, smoking, cardiometabolic biomarker, and prevalent condition data for US adults [National Health and Nutrition Examination Survey 1999-2018, NHANES: https://wwwn.cdc.gov/nchs/nhanes/continuousnhanes/default.aspx
(5) All-cause and cause-specific mortality data for US adults [National Death Index 1999-2018, NDI: https://www.cdc.gov/nchs/data-linkage/mortality-public.htm

Human research participants

Policy information about studies involving human research participants and Sex and Gender in Research.

Reporting on sex and gender	This study involved secondary data analysis for publicly available databases. No human research participants were involved in this original analysis.
Population characteristics	NA
Recruitment	NA
Ethics oversight	NA

Note that full information on the approval of the study protocol must also be provided in the manuscript.

Field-specific reporting

Please select the one below that is the best fit for your research. If you are not sure, read the appropriate sections before making your selection.

☐ Life sciences ☒ Behavioural & social sciences ☐ Ecological, evolutionary & environmental sciences

For a reference copy of the document with all sections, see nature.com/documents/nr-reporting-summary-flat.pdf

Behavioural & social sciences study design

All studies must disclose on these points even when the disclosure is negative.

Study description	Quantitative, cross-sectional and prospective analysis using secondary data from publicly available USDA and CDC databases
Research sample	Nationally representative sample of US adults, 1999-2018 (n = 47,999) from the National Health and Nutrition Examination Survey (NHANES) from 1999-2000 to 2017-2018. Study sample size was determined by NHIS staff to allow for a nationally representative sample. We included all NHANES respondents in the dietary portion of the survey, with a few exceptions listed in the "Data Exclusions" section below.
Sampling strategy	Sampling procedures including sample size calculation conducted by NHIS staff for NHANES, detailed here: https://wwwn.cdc.gov/nchs/nhanes/analyticguidelines.aspx#sample-design<br><br>Since 1999, the sample design has consisted of multi-year, stratified, clustered four-stage samples, with data release in 2-year cycles. The NHANES sample is drawn in four stages: (a) PSUs (counties, groups of tracts within counties, or combinations of adjacent counties), (b) segments within PSUs (census blocks or combinations of blocks), (c) dwelling units (DUs) (households) within segments, and (d) individuals within households. PSUs are sampled from all U.S. counties. Screening is conducted at the DU level to identify sampled persons (SPs), based on oversampling criteria. NHANES 2015–2018 oversampled

	some subgroups to increase precision for subgroup estimates.
Data collection	Data collection conducted by NHIS staff for NHANES. Secondary data for the present analysis come from the Household interview, MEC Medical Examination including two, 24-hour dietary recalls. More details provided here: https://www.cdc.gov/nchs/data/series/sr_01/sr01_056.pdf
Timing	NA. Please see specific NHANES Plan and Operation documentation for dates of data collection: https://www.cdc.gov/nchs/data/series/sr_01/sr01_056.pdf
Data exclusions	Our analysis excluded NHANES respondents without valid dietary recalls (n=7434), with extreme total energy intake (<500 or >5000 kcal/day; n=704), who only reported intake of alcoholic beverages (n=3), or with missing data on smoking status (n=34). For prospective mortality analyses, we further excluded respondents without valid linked mortality data (n=81). See Figure S1 for more details.
Non-participation	Data on response rates for NHANES surveys for each 2-year survey cycle can be found here: https://wwwn.cdc.gov/nchs/nhanes/ResponseRates.aspx#response-rates
Randomization	Participants were not allocated into experimental groups.

Reporting for specific materials, systems and methods

We require information from authors about some types of materials, experimental systems and methods used in many studies. Here, indicate whether each material, system or method listed is relevant to your study. If you are not sure if a list item applies to your research, read the appropriate section before selecting a response.

Materials & experimental systems

n/a	Involved in the study	
☒	☐	Antibodies
☒	☐	Eukaryotic cell lines
☒	☐	Palaeontology and archaeology
☒	☐	Animals and other organisms
☒	☐	Clinical data
☒	☐	Dual use research of concern

Methods

n/a	Involved in the study	
☒	☐	ChIP-seq
☒	☐	Flow cytometry
☒	☐	MRI-based neuroimaging
